# Ivermectin induces cell cycle arrest and apoptosis of HeLa cells via mitochondrial pathway

**DOI:** 10.1111/cpr.12543

**Published:** 2018-12-04

**Authors:** Ping Zhang, Yang Zhang, Kuikui Liu, Bin Liu, Wenping Xu, Jufang Gao, Lei Ding, Liming Tao

**Affiliations:** ^1^ Shanghai Key Laboratory of Chemical Biology, School of Pharmacy East China University of Science and Technology Shanghai China; ^2^ Shandong Key Laboratory of Chemical Medicine Shandong Academy of Pharmaceutical Sciences Jinan China; ^3^ Vegetable Technical Extension Station Qingpu District Shanghai Shanghai China; ^4^ College of Life and Environmental Sciences Shanghai Normal University Shanghai China

**Keywords:** antimigratory effects, apoptosis, cell cycle arrest, cervical cancer, ivermectin, mitochondrial pathway

## Abstract

**Objectives:**

The aim of study was to investigate the anticancer activities of Ivermectin (IVM) and the possible mechanisms in cells level via cell proliferation inhibition, apoptosis and migration inhibition in model cancer cell HeLa.

**Materials and methods:**

The MTT assay was used to study the inhibitory effect of IVM on the proliferation of Hela cells, and the cell cycle was analysed by flow cytometry. The neutral comet assay was used to study the DNA damage. The presence of apoptosis was confirmed by DAPI nuclear staining and flow cytometry. Changes in mitochondrial membrane potential and reactive oxygen species (ROS) levels were determined using Rhodamine 123 staining and DCFH‐DA staining. Western blot analysis for apoptosis‐related proteins was carried out. We use scratch test to analyse the antimigration potential of IVM*.*

**Results:**

Ivermectin can inhibit the viability of HeLa cells significantly. In addition, treatment with IVM resulted in cell cycle arrest at the G1/S phase which partly account for the suppressed proliferation. Typical apoptosis morphological changes were shown in IVM treatment cells including DNA fragmentation and chromatin condensation. At the same time, the results of flow cytometry analysis showed that the number of apoptotic cells increased significantly with the increase of IVM concentration. Moreover, we observed that the mitochondrial membrane potential collapses and the ratio of Bax/Bcl‐2 in the cytoplasm increases, which induces cytochrome c release from the mitochondria to the cytoplasm, activates caspase‐9/‐3 and finally induces apoptosis. We also found that IVM can significantly increase intracellular ROS content. At the same time, we determined that IVM can significantly inhibit the migration of HeLa cells.

**Conclusions:**

Our experimental results show that IVM might be a new potential anticancer drug for therapy of human cancer.

## 1 INTRODUCTION

Ivermectin (IVM) is the 22,23‐dihydro derivative of avermectin B1 and is an important member of the avermectin family of 16‐membered macrocyclic lactones evolve from the actinomycete *Streptomyces avermectinius.* 1 IVM was originally used as a veterinary drug and was very effective for a variety of parasites, such as gastrointestinal roundworms, lungworms and mites.^2^

It is believed that IVMacts on gamma‐amino butyric acid (GABA) receptor and glutamate‐gated chloride channels.^3^ Due to the low affinity for other mammalian ligand‐gated channels, IVM hardly crosses the blood‐brain barrier. Consequently, IVM has been generally supposed to be of low toxicity for the health of adults and approved for use in humans worldwide.^4^ For example, IVM was widely used for the treatment of river blindness in the form of a free drug, saving countless people’s eyesight.^5^ The discoverers of IVM were awarded the 2015 Nobel Prize in Physiology or Medicine due to it has a very successful therapeutic effect on many parasitic diseases.^6^ In the recent years, IVM has been reported to exert anticancer activities. However, the specific roles of IVM in cancer remain unclear.

Cancer is a group of diseases with characteristics involving abnormal and uncontrolled cell growth, the potential for penetration and diffusion to other parts of body, considered as the leading cause of death in the world.^7^ Therefore, the research of antitumour drugs with high efficiency and low toxicity has been the focus of drug discovery.^8^ The HeLa cell line derived from cervical cancer cells was the first successful attempt to immortalize human‐derived cells in vitro and has served a role analogous to that of a model organism due to its robust growth and unrestricted distribution.^9^ Cervical cancer is one of the most common malignancy throughout the world which has become the second female malignant tumour, and has a serious threat to the health and safety of women.^10^ Based on these two points above, we chose to use HeLa cells for our study.

Ivermectin has been demonstrated to have anticancer activities on various types of cancer including glioblastoma, chronic myelogenous leukaemia and breast cancer.^11^, ^12^, ^13^ Because of the lack of systematic research on the anticancer activity of IVM in HeLa cells, the aim of this study was to investigate the activity and underlying mechanism of IVM against HeLa cells, and to provide experimental basis and theoretical foundation for further research on theory and clinical study.

## 2 MATERIALS AND METHODS

### 2.1 Chemicals and antibodies

Ivermectin (95.9% purity) was purchased from (Cato Research Chemicals Inc., Eugene, OR, USA) 3‐(4,5‐Dimethylthiazol‐2‐yl)‐2,5‐diphenyl‐tetrazolium bromide (MTT), Rhodamine123 (Rh‐123), 4′6‐diamidino‐2‐phenylindole (DAPI), N,N,N′,N′‐tetramethylethylenediamine, dimethylsulfoxide (DMSO), phenylmethanesulfonyl fluoride (PMSF), 2′,7′‐Dichlorodihydrofluorescein diacetate (DCFH‐DA), propidium iodide (PI), RIPA lysis buffer were purchased from Sigma (Beverly, MA, USA). IVM is dissolved in DMSO and diluted to the appropriate concentration in using cell culture medium. It should be noted that the final concentration of DMSO in the cell culture medium should be less than one thousandth. Antibodies such as cytochrome c, caspase‐9, caspase‐3 were purchased from Cell Signaling Technology (Beverly, MA, USA). Bax, Bcl‐2, PARP, p53, CDK4 and CyclinE were brought from Sangon (Shanghai, China). Secondary anti‐rabbit antibody was from Sangon Biotech Co. Ltd. Other common chemicals and reagents were purchased from Shanghai Titan‐chem Co. Ltd. (Shanghai, China).

### 2.2 Cell culture

HeLa cell line (Human cervical carcinoma) was acquired from ATCC and grown in DMEM medium (Hyclone, Logan, UT, USA) supplemented with 10% FBS (Gibco, USA) and 1% penicillin/streptomycin (Hyclone) in an incubator with a humidified air atmosphere of 5% CO_2_ at 37°C.

### 2.3 Cell viability assay

The MTT colorimetric method is a method that can quickly quantify the number and the value‐added state of living cells.^14^ It is widely used for cytotoxicity detection due to its advantages such as small workload, easy operation, rapidity and good repeatability.^15^ The HeLa cells (5 × 10^3^ cells/well) were seeded onto 96‐well plates. After 24 hours of incubation, cells were treated with the corresponding concentration (0, 2.5, 5, 7.5, 10 and 20 μmol/L) of IVM for 24 and 48 hours. Complete DMEM medium was used as a blank. Four hours prior to the assay, MTT reagent (20 μL/well, 5 mg/mL) was added to cells. We replaced the medium with DMSO (200 μL/well), and then we read the absorbances at 492 and 630 nm by a microplate reader (Bio‐Teck, Winooski, VT, USA).

### 2.4 Cell cycle analysis

The HeLa cells (1 × 10^6^ cells/well) were seeded onto 9.6 cm^2^ plates overnight and treated with specified concentrations (0, 2.5, 5, 10 and 20 μmol/L) of IVM. Then, the cells were harvested and resuspended in PBS and fixed with 70% ethanol and left at − 20°C overnight. After 12 hours of fixation, cells were centrifuged at 600 *g* for 5 minutes, washed and resuspended in PBS (200 μL). Then, we added 100 μL propidium iodide solution (500 μg/mL) to the cells and incubated for 15 minutes in the dark.^16^ The cell cycle was then analysed by flow cytometry (BD FACS Calibur). Data were analysed by the flowjo software.

### 2.5 DNA damage assay

The neutral comet assay was used to study the DNA damage induced by IVM.^17^ Briefly, HeLa cells (1 × 10^5^ cells/well) were incubated overnight, and treated with different concentration (0, 2.5, 5, 10 and 20 μmol/L) of IVM for 12 hours. Then, the cells were harvested and suspended in PBS. Subsequently, the cell suspension was mixed with 1% normal melting agarose and rapidly added to a previously prepared 0.8% normal melting agarose slide. After solidification, the slides were immersed into the lysis buffer for 2 hour at 4°C in the dark. The slides were immersed in electrophoresis buffer (1 × TAE [Tris base, Acetic acid and EDTA]) in the electrophoresis tank. After 10 minutes, we applied 20 V or 300 mA electric field for 10 minutes. After the slide was washed twice with ddH_2_O, 20 μL PI solution was added. Cell images were captured with a fluorescence microscope (Lecois, DM3000, GER) and analysed using the imagej software.

### 2.6 Chromatin condensation detection

HeLa cells were seeded (1 × 10^5^ cells/well) in a 12‐well plate and incubated overnight. Then, the cells were exposed to IVM (0, 2.5, 5, 10 and 20 μmol/L) for 24 hours. After fixed with paraformaldehyde, the cells were stained by DAPI (1 μL/well, 1 μg/mL) for 15 minutes at 37°C. Changes in nuclear morphology induced by IVM were observed using fluorescent microscopy.

### 2.7 Flow cytometry analysis of cell apoptosis

Apoptosis rate was determined using the Alexa Fluor 488 Annexin V/DeadCell Apoptosis Kit. Briefly, after HeLa cells (1 × 10^6 ^cells/well) were exposed to IVM (0, 2.5, 5, 10 and 20 μmol/L) for 24 hours, cells were centrifuged at 100 *g* for 5 minutes, washed and suspended in PBS. Then, cells were labelled with Annexin V‐FITC and PI for 20 minutes. Finally, apoptosis rate was determined by flow cytometer (FACS Calibur, BD, San Jose, CA, USA) and flowjo software.

### 2.8 Mitochondrial membrane potential (Δ*Ψ*m) analysis

The effect of IVM on mitochondrial membrane potential was assessed using fluorescence microscopy. HeLa cells were seeded (1 × 10^5^ cells/well) in a 12‐well plate and incubated overnight. After HeLa cells were treated with 0, 2.5, 5, 10 and 20 μmol/L IVM for 6 hours, cells were stained with Rhodamine123 for 30 minutes at 37°C. The phenomenon of cells was photographed by fluorescence microscopy. The images were measured using imagej software.

### 2.9 Western blotting

After treating HeLa cells (1 × 10^6^ cells/well) with 0, 2.5, 5, 10 and 20 μmol/L IVM for 6 hours, the cells were collected, washed with PBS and lysed with RIPA buffer. Protein quantification was performed using BCA Protein Assay Kit. Equal amounts of protein were loaded onto an SDS‐PAGE gel for electrophoresis and transferred to polypropylene difluoride (PVDF) membranes. After blocking with 5% nonfat milk for 2 hours, the membranes were incubated with the primary antibody for 12‐16 hours. The membranes were then incubated with secondary antibody for 2 hours at room temperature. And the change in target protein expression was detected by a chemiluminescent gel imaging system (Tanon, Shanghai, China).

### 2.10 Intracellular ROS measurement

The effect of IVM on intracellular reactive oxygen species was evaluated by fluorescence microscopy.^18^ HeLa cells were seeded (1 × 10^5^ cells/well) in a 12‐well plate and incubated overnight. After HeLa cells were treated with 0, 2.5, 5, 10 and 20 μmol/L IVM for 6 hours, cells were tinted with oxidation of DCFH‐DA for 30 minutes at 37°C. The phenomenon of cells was photographed by fluorescence microscopy. The images were measured using imagej software.

### 2.11 Antimigration assay

We use scratch test to analyse the antimigration potential of IVM. HeLa cells (1 × 10^5^ cells/well) were seeded in 6‐well plate. When the cell density reaches about 70%, we use a 200 μl yellow pipette tip to draw a straight line in the cell culture plate.^19^ Since antimetastatic agents usually require long‐term use by patients, they should have low cytotoxicity. Therefore, in the current study, we have used IVM at lower concentrations than that used for other assays (2.5, 5, 10, 15 μmol/L). After scratching, HeLa cells were exposed to different concentrations of IVM for 48 hours. The images of cell migration were captured using fluorescence microscopy. The data analysis was performed using imagej soft‐ware.

### 2.12 Data analyses

At least three independent biological replicates were performed in all experiments. Data were shown as mean ± standard deviation. To determine differences between groups, we performed the Dunnett test using ANOVA (*P* < 0.05 indicates statistical significance). spss 17.0 (SPSS Inc., Chicago, IL, USA) was used for all statistical analyses.

## 3 RESULTS

### 3.1 Inhibition of HeLa cells viability by IVM

**Figure 1:**
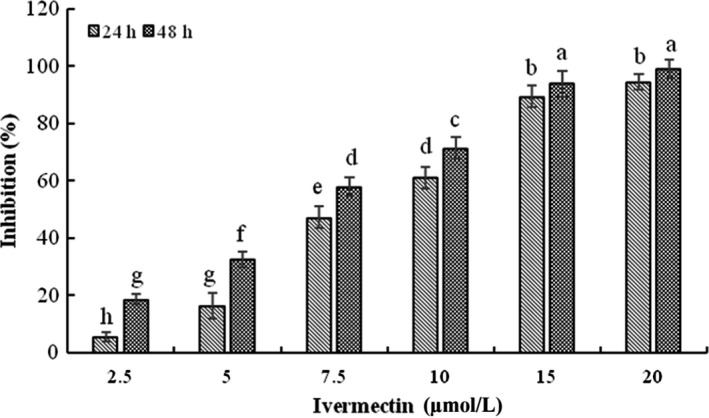
Cytotoxicity of IVM on HeLa cells. Cell viability of HeLa cells treated with various concentrations (2.5, 5, 7.5, 10, 15 and 20 μmol/L) IVM for 24 and 48 h. Different small letters show significant differences between any two groups (*P* < 0.05). The data are shown as the means ± SD of three independent experiments. IVM, ivermectin

To evaluate the cytotoxic of IVM on HeLa cells, we analysed the cell viability using the MTT assay. IVM can significantly inhibit the activity of HeLa cells in a time‐ and dose‐dependent manner (Figure 1). The proliferation inhibitory ratios of HeLa cells were 5.67% ± 1.83%, 16.39% ± 2.75%, 48.37% ± 4.25%, 61.15% ± 3.82%, 89.52% ± 4.47%, 95.35% ± 3.12% after 24 hours of treatment with IVM at the different concentrations of 2.5, 5, 7.5, 10, 15, 20 μmol/L. After 48 hours treatments, the proliferation inhibitory ratio reached to 18.64% ± 1.61%, 32.73% ± 4.35%, 55.36% ± 3.67%, 71.45% ± 3.92%, 95.9707% ± 3.28%, 99.049 88% ± 2.18%. IC_50_ values for IVM are provided in Table 1.

**Table 1:** The half maximal inhibitory concentration (IC_50_) of HeLa cells exposed to IVM

Cell lines	Treatment time (h)	*a* (intercept)	*b* (slope)	IC50 (μmol/L)	95% (confidence limits)	
					Lower	Upper
HeLa	24	2.42	1.19	7.87 a	7.16	8.51
	48	2.28	2.08	5.78 b	5.13	6.95

IVM, ivermectin.

Small alphabet indicated statistically significant differences between two groups in the same column (*P* < 0.05).

### 3.2 IVM‐induced cell cycle arrest at G1/S phase

**Figure 2:**
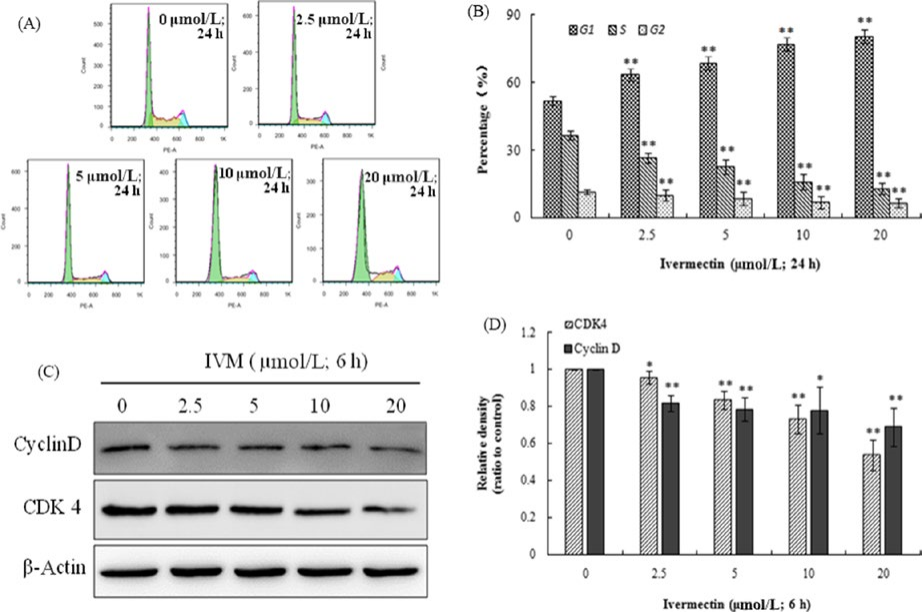
Analysis of cell cycle by flow cytometry (A). The percentage of cells in G1, G2 and S phase in HeLa cells without or with the treatment of IVM for 24 h (B). Cyclin D expression and CDK 4 expression are both decreased as the IVM concentrations increased (C). The densitometric analysis results of Cyclin D and CDK4 were shown in the right panel (D). Data were calculated by mean ± SD of three independent experiments. *P < 0.05, **P < 0.01 indicate the significant differences from the control. IVM, ivermectin

Flow cytometry of propidium iodide staining was performed to analyse the cell cycle distribution. As is shown in Figure 2A, cells treated with different concentrations of IVM for 24 hours showed a significant increase in the number of cells in the G0/G1 phase, which corresponds to a significant decrease in the number of cells in the S and G2/M phases (*P < 0.05, **P < 0.01). Further Western Blot results showed that Cyclin D and CDK4 expressions were significantly reduced (Figure 2B).

### 3.3 IVM‐induced DNA damage in HeLa cells

**Figure 3:**
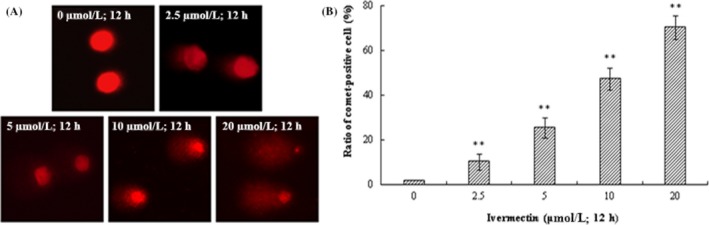
IVM induced DNA damage in HeLa cells. DNA fragments were shown as comet images in alkaline gel electrophoresis (200×; A). Percentage of comet‐positive cells in the treatment of IVM for 12 h analysis results were shown in the right panel (B). The data are shown as the means ± SD of three independent experiments. **P < 0.01. IVM, ivermectin

We used single‐cell gel electrophoresis (comet assay) to evaluate the DNA damage induced by IVM. During electrophoresis, the broken DNA fragments move towards the positive electrode by carrying a negative charge, and the unbroken DNA does not move. After staining, the nucleus forms a bright head, and the DNA fragments form a tail, which resembles a comet‐like shape. The more severe the DNA damage, the longer the tail length. It can be seen from the Figure 3A that the nuclear chromatin DNA of the untreated control group of IVM is basically not broken, the nucleus is intact, and it is round, and no obvious comet‐like tail is observed. However, the nuclear chromatin DNA of the treatment group is obviously broken and formed a long comet‐like tail, the "tailing" phenomenon. The percentages of comet‐positive cells of HeLa increased significantly after treated by IVM in a dose‐dependent way (Figure 3B). The distributions of IVM‐treatment HeLa cells, with respect to comet assay parameters including tail lengths and tail DNA are presented in Table 2 (**P < 0.01). The results showed that there was no obvious DNA damage in the untreated control group, and the DNA damage of HeLa cells was significantly improved after IVM treatment.

**Table 2:** The parameters of DNA damage in HeLa cells exposed to the different concentrations of IVM for 12 h in alkaline comet assay

Concentrations (μmol/L)	Comet assay parameters	
	Tail DNA (%)	Tail length (μm)
0	1.78 ± 0.15 e	1.39 ± 0.57 d
2.5	10.27 ± 3.255 d	3.96 ± 1.49 d
5	25.48 ± 4.57 c	11.75 ± 3.43 c
10	47.32 ± 5.18 b	21.16 ± 5.31 b
20	70.12 ± 5.36 a	30.46 ± 3.92 a

IVM, ivermectin.

The data are shown as the means ± SD of three independent experiments.

Small alphabet indicated statistically significant differences between any two groups in the same column (*P* < 0.05).

### 3.4 IVM‐induced apoptosis in HeLa cells

**Figure 4:**
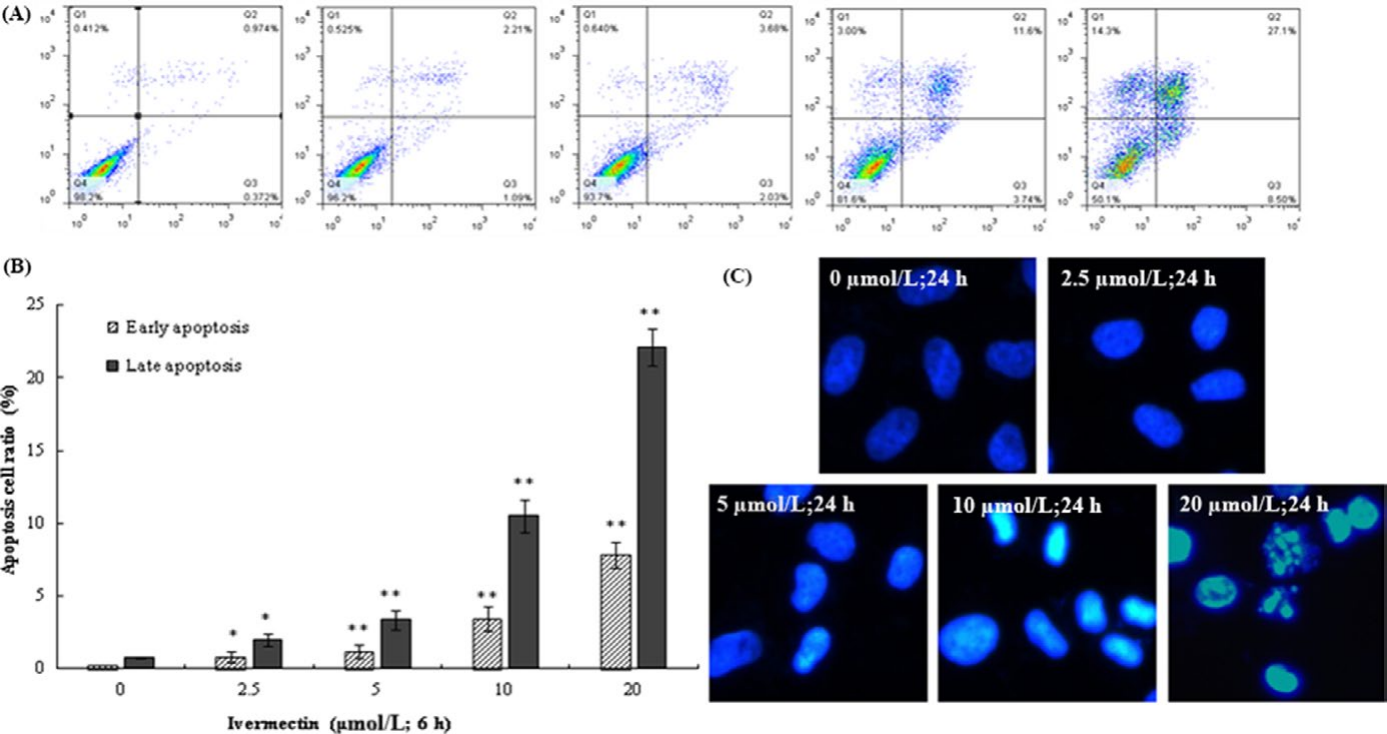
IVM induced apoptosis in HeLa cells. The lower left panel shows the normal cells, the lower right panel shows the early apoptotic cells and the upper right panel shows the late apoptotic cells or undergoing necrotic cells (12 h) (A). The apoptosis cells ratio was shown in the right panel (B). Cell nuclei were observed by fluorescence microscopy (200×). Typical apoptosis morphological changes were shown in treated cells including chromatin condensation and DNA fragmentation (C). The data are shown as the means ± SD of three independent experiments. *P < 0.05, **P < 0.01. IVM, ivermectin

After treatment with different concentrations of IVM for 24 hours, IVM‐induced apoptosis was measured by flow cytometry (Figure 4A). The different labelling patterns in the annexin V/PI analysis were used to identify the different cell populations where FITC‐negative and PI‐negative cells were designated as viable cells. FITC‐positive and PI‐negative cells were identified as early apoptotic cells. FITC‐positive and PI‐positive cells were identified as late apoptotic cells. FITC‐negative and PI‐positive cells were identified as necrotic cells. The results showed that the proportion of early apoptosis cells increased from 0.231% ± 0.15% in the control cells to 0.818% ± 0.24%, 1.19% ± 0.37%, 3.46% ± 0.35% and 7.81% ± 0.63% and the proportion of late apoptosis cells added from 0.231% ± 0.33% in the control cells to 1.97% ± 0.42%, 3.38% ± 0.65%, 10.5% ± 1.15% and 22.1% ± 1.27% in 2.5, 5, 10 and 20 μmol/L. IVM‐treated cells, separately (*P < 0.05, **P < 0.01). The results showed that IVM had a concentration‐dependent effect on apoptosis effect of HeLa cells (Figure 4B). In order to further verify the effect of IVM on the nucleus of cervical cancer cells, the nuclear dye DAPI was used for staining observation. As the concentration of IVM increases, the nucleus of HeLa cells undergoes typical morphological changes such as nuclei condensing, fragmentation (Figure 4C).

### 3.5 IVM‐induced mitochondrial membrane potential collapse in HeLa cells

**Figure 5:**
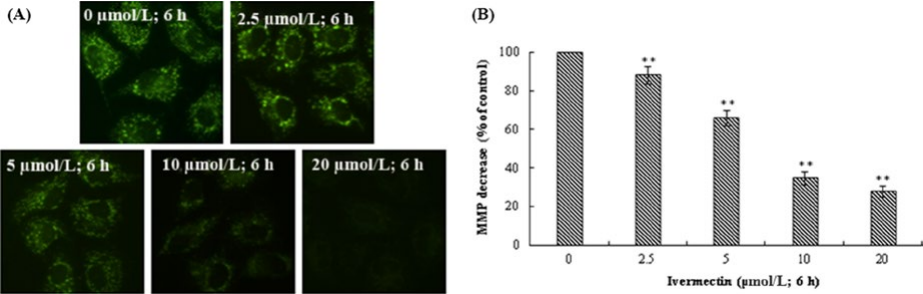
IVM induced MMP collapse in HeLa cells. The fluorescence intensity of Rh‐123 in mitochondria was examined by fluorescent microscopy (200×) (A). The data of MMP decrease were shown in the right panel (B). The data are shown as the means ± SD of three independent experiments. **P < 0.01. IVM, ivermectin; MMP, mitochondrial membrane potential

Mitochondrial membrane potential (*MMP*) is one of the important indicators for maintaining normal mitochondrial configuration and function.^20^ Decreased *MMP* is one of the important factors leading to apoptosis and is considered to be the first step of the apoptosis cascade.^21^ Therefore, we tried to evaluate the influence of IVM on *MMP *in HeLa cells. We found that exposure of HeLa cells to different concentrations (0, 2.5, 5, 10 and 20 μmol/L) of IVM for 6 hours resulted in a gradual decrease in green fluorescence intensity (Figure 5A,B). The study shows that the decrease of *MMP *was brought about by IVM treatment in HeLa cells (**P < 0.01).

### 3.6 Effect of IVM on the expression of apoptosis‐related proteins in HeLa cells

**Figure 6:**
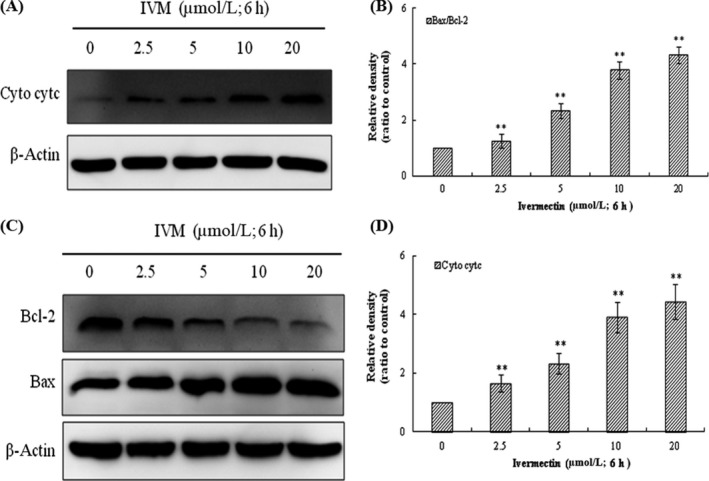
Influence of IVM on the level of apoptosis‐associated proteins in HeLa cells. IVM induced the release of cytochrome c into the cytosol in a dose‐dependent manner (A). Cyto denotes cytosolic fractions. Bax expression increased and Bcl‐2 expression decreased as the IVM concentrations increased (C). The densitometric analysis results of Cyto cytc and Bax/Bcl‐2 were shown in the right panel (B and D). β‐actin was used as an equal loading control. The data are shown as the means ± SD of three independent experiments. **P < 0.01. IVM, ivermectin

The role of mitochondria in the mechanism of apoptosis has attracted more and more attention. On the one hand, cytochrome c released into the cytoplasm of the mitochondrial membrane gap triggers the caspase cascade, leading to cell death; on the other hand, the Bcl‐2 protein family regulates the release of cytochrome c from mitochondria. Therefore, cytochrome c is a key factor in the apoptosis signalling process in mammals. To determine the underlying mechanism of IVM‐induced apoptosis, we tested the expression of apoptosis‐related proteins such as cytochrome c, Bax and Bcl‐2 in HeLa cell. Cytochrome c content in cytoplasm was increased in a dose‐dependent manner in IVM‐induced HeLa cells (Figure 6A,B). Meanwhile, the expression level of anti‐apoptosis protein Bcl‐2 was down‐regulated and pro‐apoptotic protein Bax was elevated in a dose‐dependent manner (Figure 6C,D; **P < 0.01).

### 3.7 Effect of IVM on caspase‐3, caspase‐9,P53 and PARP in HeLa cells

**Figure 7:**
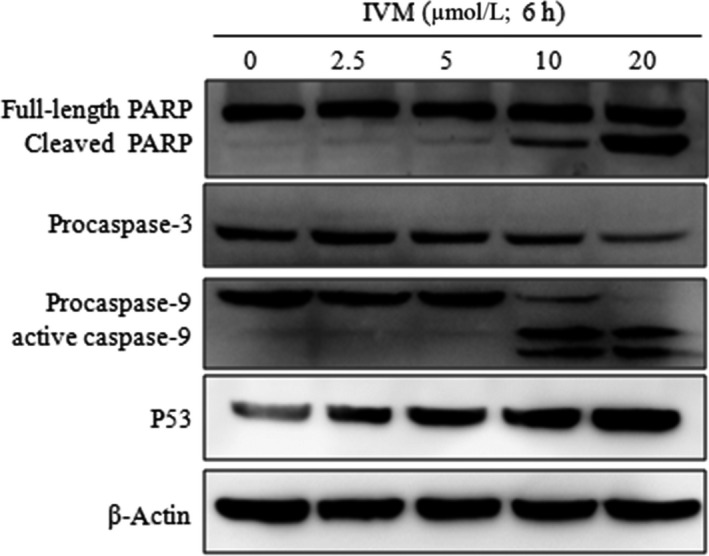
Influence of IVM on the expression levels of the PARP, procaspase‐3, caspase‐9 and P53 in HeLa cells. HeLa cells were treated with various concentrations (0, 2.5, 5, 10 and 20 μmol/L) IVM for 6 h. IVM, ivermectin

The process of apoptosis involves a complex cascade of proteolytic hydrolysis.^22^ The Caspase family plays an extremely important role in the process of apoptosis.^23^ P53 is a tumour suppressor protein that plays an important role in the mitochondrial apoptosis pathway.^24^ To further investigate the apoptosis of HeLa cells caused by IVM, we examined the activity of caspase‐3, caspase‐9, p53 and PARP by Western blot assay. As shown in Figure 7, caspase‐3 and caspase‐9 were both activated while cleaved PARP increased and P53 protein expression was significantly increased.

### 3.8 IVM‐induced generation of intracellular ROS in HeLa cells

**Figure 8:**
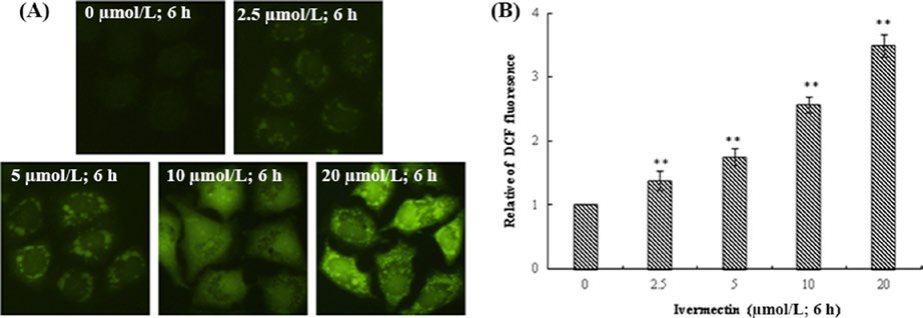
IVM induced generation of intracellular ROS in HeLa cells. The treated cells were collected for DCFH‐DA fluorescence was examined by fluorescent microscopy (200×) (A). The data of ROS generation were shown in the right panel (B). The data are shown as the means ± SD of three independent experiments. **P < 0.01. IVM, ivermectin

We analysed the intracellular reactive oxygen species (ROS) level using fluorescence microscopy after DCFH‐DA being hatched in cells. Intracellular ROS of HeLa cells were significantly increased after treatment with different concentrations of IVM (2.5, 5, 10 and 20 μmol/L) compared to the 0 μmol/L IVM group (Figure 8A). The studies suggested ROS increases in a dose‐dependent way in IVM‐treated HeLa cells (Figure 8B; **P < 0.01).

### 3.9 Migration of HeLa Cells was Inhibited by IVM

**Figure 9:**
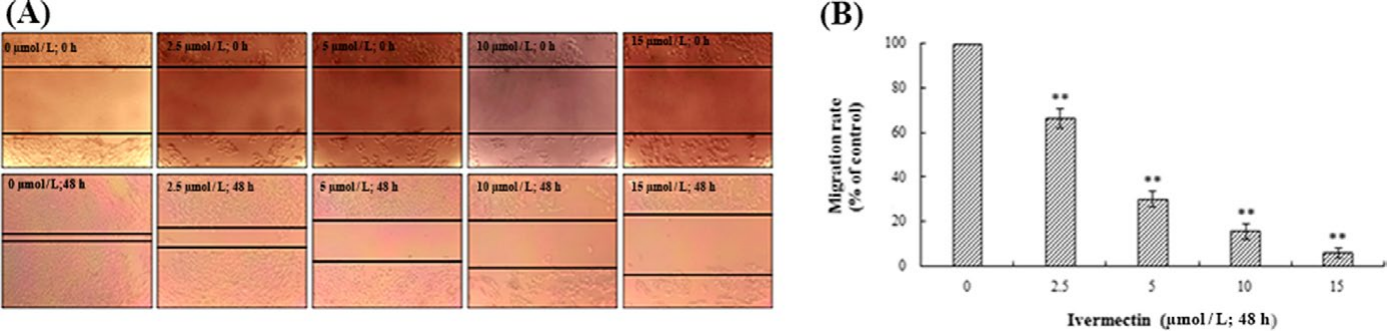
Analysis of migration of HeLa cells in vitro. Cells in 6‐well plates were wounded by scratching them with a pipet tip, and the cells were incubated with IVM (0, 2.5, 5, 10 and 15 μmol/L) for 48 h. The cells were photographed under phase‐contrast microscopy (5 × magnification) (A). The data of migration rate were shown in the right panel (B). Data were calculated by mean ± SD of three independent experiments. **P < 0.01. IVM, ivermectin

We further verified the inhibitory effect of IVM on HeLa cell migration using antimigration assay (Figure 9A). Since higher concentrations of IVM may cause apoptosis, this study was performed at lower concentrations to confirm the antimigratory potential. After 48 hours of incubation, the cells in the control group gradually began to heal and grow into dense cell clusters, and in cells treated with IVM (2.5, 5, 10 and 15 μmol/L), we observed that only a few cells grew to the internal space of the wound as the concentration of IVM was increased (**P < 0.01).

## 4 DISCUSSION

In recent years, the use of bacterial or fungal fermentation to prevent and treat cancer is attracting more and more attention.^25^ IVM is a semi‐synthetic macrocyclic lactone derivative of the avermectin family.^26^ Nowadays, because of it has no side effects on human safety, IVM has been approved for use in humans to treat on chocerciasis and lymphatic filariasis.^27^ Accumulating evidence suggests that IVM has anticancer activities against breast cancer, ovarian cancer, neurofibromatosis and chronic myeloid leukaemia. In our study, we investigated the effects of IVM on cervical cancer (HeLa) cell line and explored the underlying mechanisms.

To evaluate the cytotoxicity of IVM on HeLa cells, we used the MTT assay to assess the effects of different concentrations of IVM on the viability of HeLa cells. The results showed that IVM has a significant inhibitory effect on theproliferation of HeLa cells. Meanwhile, the result of alkaline comet assay analysis indicated that IVM inducing DNA fragmentation in HeLa cells. As we all know, DNA damage can induce inactivation of the CDK‐Cyclin complex, resulting in cell cycle arrest.^28^ Cell cycle progression is one of a pivotal signalling mechanisms of homoeostasis maintenance in healthy tissues and normal cells.^29^ Therefore, induction of cell arrest in cancer cells is one of the useful strategies for anticancer drug development.^30^ Cell cycle analysis shows that IVM induces G1/S cycle arrest in HeLa cells, which means that IVM inhibits proliferation of HeLa cells via cell cycle arrest resulting from DNA damage.

It is well known that three pathways can lead to cell death, namely apoptosis, autophagy and cell necrosis.^31^ At present, targeting apoptosis is the most successful way to treat cancer in addition to surgery. We further designed experiments to explore whether IVM can cause apoptosis in HeLa cells. Through DAPI staining, we clearly observed the obvious morphological features of apoptosis such as cell shrinkage, chromatin condensation and DNA fragmentation.^32^ As shown in the flow cytometry, IVM could induce the increase of apoptosis rate of HeLa cells in a dose‐dependent manner which further confirmed the apoptosis effect of IVM.

As we all know, the apoptosis pathway is divided into two major types: intrinsic pathway (mediated by mitochondria) and the extrinsic pathway (mediated by death receptors).^33^ In order to identify the type of IVM‐induced apoptosis effect, we observed the change of intracellular mitochondrial membrane potential by rhodamine 123 staining. The results showed that IVM caused the collapse of mitochondrial membrane potential. Moreover, the Western blot experiment shows that the content of cytochrome c in cytoplasm increases obviously. Cytochrome c released from mitochondria to the cytoplasm is the most important event in mitochondrial mediated apoptosis signal transduction pathway.^34^ Therefore, we have determined that IVM induces apoptosis in HeLa cells through mitochondrial pathways. We also examined the expression levels of Bcl‐2 family proteins and caspase‐3/‐9 proteins. The activated caspase cleaves PARP, a substrate protein of caspase‐3, which is one of the hallmarks of apoptosis. Results as we expected, IVM inhibited the expression of apoptosis protein Bcl‐2, increased the expression of apoptosis protein Bax. And further activated the downstream apoptosis‐inducing caspase‐3/‐9, and cleaved PARP which eventually led to apoptosis. It is well known that intracellular ROS are involved in the regulation of apoptosis, cell cycle and participate in various signal transduction pathways in cells. We found that IVM can significantly increase intracellular ROS content.

Rapid metastasis is one of the biggest malignant features of cancer cells.^35^ The invasion and metastasis of malignant tumours are a series of complex and multi‐step interactions.^36^ It involves the malignant proliferation of cancer cells, the adhesion of tumour cells, movement, invasiveness and the ability to metastasize. Tumour invasion is the premise of cancer metastasis.^37^ Invasion does not mean that metastasis must occur. However, tumour metastasis must invade.^38^ We determined that IVM can significantly inhibit the migration of HeLa cells by the scratch test.

In conclusion, we have demonstrated that IVM can significantly inhibit the proliferation of HeLa cells via cell cycle arrest resulting from DNA damage, and induced apoptosis of HeLa cells through the mitochondrial pathway. At the same time, we found that IVM can inhibit its migration activity significantly. Through these studies, we determined that IVM has significant anticancer activity against HeLa cells. However, the study of the effect of IVM on cancer in vivo has not yet been carried out. This will be the focus of our future work.

## CONFLICT OF INTEREST

The authors declare no conflict of interest.
